# SURFACE ELECTROMYOGRAPHY OF MASSETER AND TEMPORAL MUSCLES WITH USE PERCENTAGE WHILE CHEWING ON CANDIDATES FOR GASTROPLASTY

**DOI:** 10.1590/0102-6720201600S10013

**Published:** 2016

**Authors:** Andréa Cavalcante dos SANTOS, Carlos Antonio Bruno da SILVA

**Affiliations:** Ceara Obese Nucleus, Fortaleza, CE, Brazil

**Keywords:** Electromyography, Mastication, Gastroplasty, Speech, language and hearing sciences

## Abstract

**Background:**

Surface electromyography identifies changes in the electrical potential of the muscles during each contraction. The percentage of use is a way to treat values enabling comparison between groups.

**Aim:**

To analyze the electrical activity and the percentage of use of masseter and temporal muscles during chewing in candidates for gastric bypass.

**Methods:**

It was used Surface Electromyography Miotool 200,400 (Miotec (r), Porto Alegre/RS, Brazil) integrated with Miograph 2.0 software, involving patients between 20-40 years old. Were included data on electrical activity simultaneously and in pairs of temporal muscle groups and masseter at rest, maximum intercuspation and during the chewing of food previously classified.

**Results:**

Were enrolled 39 patients (59 women), mean age 27.1+/-5.7. The percentage of use focused on temporal muscle, in a range of 11-20, female literacy (n=11; 47.82) on the left side and 15 (65.21) on the right-hand side. In the male, nine (56.25) at left and 12 (75.00) on the right-hand side. In masseter, also in the range of 11 to 20, female literacy (n=10; 43.48) on the left side and 11 (47.83) on the right-hand side. In the male, nine (56.25) at left and eight (50.00) on the right-hand side.

**Conclusion:**

40-50% of the sample showed electrical activity in muscles (masseter and temporal) with variable values, and after processing into percentage value, facilitating the comparison of load of used electrical activity between the group, as well as usage percentage was obtained of muscle fibers 11-20% values involving, representing a range that is considered as a reference to the group studied. The gender was not a variable.

## RESUMO

**Racional:**

A eletromiografia de superfície identifica variações dos potenciais elétricos dos músculos durante cada contração realizada. O percentual de uso é uma forma de tratar valores possibilitando comparação entre grupos.

**Objetivo:**

Analisar a atividade elétrica e o percentual de uso dos músculos masséteres e temporais durante a mastigação em candidatos à gastroplastia.

**Métodos:**

Utilizado Eletromiógrafo de Superfície Miotool 200/400 (Miotec^(r)^, Porto Alegre/RS, Brasil) integrado ao software Miograph 2.0 em pacientes entre 20-40 anos candidatos à gastroplastia. Foram incluídos dados relativos à atividade elétrica simultânea e em pares dos grupos musculares temporais e masséteres em repouso, máxima intercuspidação habitual e durante mastigação de alimento previamente classificado.

**Resultados:**

Foram analisados 39 pacientes (59% feminino), média de idade de 27,1+5,7. O percentual de uso se concentrou em musculatura temporal, em faixa de 11-20% no sexo feminino (n=11; 47,82%) referente à lateral esquerda e 15 (65,21%) na lateral direita. No masculino, nove (56,25%) na lateral esquerda e 12 (75,00%) na lateral direita. Em masséteres, também na faixa de 11-20% no sexo feminino (n=10; 43,48%) referente à lateral esquerda e 11 (47,83%) na lateral direita. No masculino, nove (56,25%) na lateral esquerda e oito (50,00%) na lateral direita.

**Conclusão:**

Entre 40-50% da amostra houve variação na atividade elétrica em masséteres e temporais, e, após transformação em valor percentual, facilitou-se a comparação da carga de atividade elétrica utilizada entre o grupo, bem como obteve-se percentual de uso de fibras musculares envolvendo valores de 11-20%, representando uma faixa que se considera como referencial para o grupo estudado. O gênero não constituiu variável em pacientes obesos mórbidos.

## INTRODUCTION

Mastication is the physiological exercise involving all senses^22^ ^,^ ^23^ being considered an important function of the stomatognatic system^14^. The structures involved in the masticatory act responsible for maintaining healthy conditions for the execution of this function, are considered fixed and mobile serving as the basis of the movements and forces involved in this context^5^.

The process involving the masticatory function is developed in three stages: 1) incision, where food is seized and cut in the region of incisors (phase which lasts from 5-10% of mastication), after being brought to the region premolars for 2) crunch (65-70% of mastication) and 3) molar, spray, generating interocclusal pressure, with the milling of food during chewing stroke (25-30% of the masticatory act)^5^.

During mastication, the muscles responsible for this function, especially the masseter, the temporal and the buccinator provides crushing force of great importance in the mastication cycles^18^ ^,^ ^20^ as well, it is necessary that the concentration of muscle movement is assembled in premolar and molar regions^23^.

Several studies advocate muscle function responsible for masticatory act with the goal of efficiency for good nutrition^3^ ^,^ ^7^ ^,^ ^23^ and, despite considering this area of orofacial motricity in its relation with speech therapists in bariatric surgery team, a field new insert, contains no proof of their efficiency^6^, but is sought tirelessly this purpose throughout the scientific research.

Explaining a little more about the muscle function, are verifiable electric potential with current technology. According to the study, this electricity is based on the principle of cell ability to trigger electrical activity through the existence of electric potential between their plasma membranes with the presentation of electrically negative cytoplasm in relation to the extracellular^26^.

A muscle or muscle group to be stimulated to move in activity, there is modification of the resting potential, turning into action potential that through electromyographic reading, presents the possibility of counting increasingly objetive^26^.

Surface electromyography (EMG'S) is a form of measurement of the masticatory muscles functionality able to identify variations of the electric potential of the muscles during each performed contraction, supporting the development of diagnostic and therapeutic functions and orofacial motor disorders, both in chewing and swallowing. Its accuracy in the records of the electrical activity of a muscle or a muscle group is what gives this technology its applicability in bigger scale^1^ ^,^ ^2^ ^,^ ^14^ ^,^ ^21^ ^,^ ^26^.

As the electromyographic signal presents great variability in comparison process to different records for the same individual or different individuals, it sought a way to compare a group of people with similarities in their sign. Normalization techniques allow comparison of electromyographic signal values, allowing the study inter individual^1^.

Through searching the literature, was observed the use of EMG'S in speech therapy; however, little was found specifically in the surgical treatment of obesity, where one realizes that more people are experiencing this procedure because it is something more effective maintenance of weight lost over a prolonged period^15^.

This study aimed to analyze the electrical activity of the masseter and temporal muscles with a percentage of use, during mastication in morbidly obese patient's candidates for gastroplasty.

## METHODS

It is the study of quantitative, transversal and descriptive approach. The collection period was from October/2012 to March/2013. The project was approved by the Ethics Committe of the University of Fortaleza under No. 114,609/2012.

The subjects of the research were selected by convenience and voluntarily in the Núcleo do Obeso do Ceará, located in Fortaleza, CE, Brazil.

Were used as inclusion criteria patients aged between 20-40 years old and candidates for gastroplasty; and the exclusion of those who presented facial and/or occlusal deformity that prevented the implementation of mastication collection.

It was used for the measurements of electrical activity of the surface electromyography apparatus Miotool 200/400 - (Miotec(r), Porto Alegre / RS, Brazil) with four channels, SDS500 sensor and integrated Miograph 2.0 software.

The technique used and the guidelines were previously explained to the patient for data collection protocols already published^9^ ^,^ ^14^ ^,^ ^16^ ^,^ ^23^ ^,^ ^26^. During habitual mastication, it was standardized as food, a portion of french bread 5 cm^3^ and signal capture time during chewing; they were analyzed in all ranges without exclusions, taking advantage of the total sample time until swallowing food. Patients underwent mastication with own cycles and spontaneous swallowing.

All EMG'S tests were performed by the same observer (SAC) and under the same environmental conditions.

Prior to each sampling site friction with non-sterile gauze soaked in 70% alcohol was performed in order to minimize artifacts^14^ and improve signal capture. The reference electrode (earth) was placed in the front portion of the patient's head.

Data were obtained on the simultaneous electrical activity and pairs of temporal and masseter muscle groups during the tasks: rest, maximum clenching in maximum habitual intercuspation (MHI) and during the chewing food period.

The records were collected by MHI maintained for 5 seconds and repeat three times with 1 min interval addition to rest between each collection and used the average for signal normalization, equivalent to 100% of the electrical activity. The signals collected during mastication were analyzed by Root Mean Square (RMS) and expressed in microvolts (uV)^9^ ^,^ ^14^ ^,^ ^26^.

To analyze the percentage of use (PU), the following formula was used:



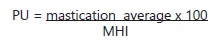



The PU is a way of normalizing the percentage of use of the muscles in activity, treated within the study group, to allow comparison of results.

### Statistical analysis

In the analysis and interpretation of the data was used descriptive statistics with the use of measures of central tendency, represented by the mean, standard deviation and the maximum and minimum values. For the purposes of the calculation was used the Excel software v. 2010 (Microsoft, 2010). Elected 39 patients and planning involving the age group occurred in order to make it more concise and reduced in certain muscular condition with the goal of much more homogeneous group, as with the range of age and very real differences, disaggregates the condition comparison of muscle activity. For this fact, the increasing age influences the muscle pattern modification and aging can change aspects related to tonicity, influencing the obtained results ^21^.

## RESULTS

The group effectively studied consisted of 39 patients, 23 (59%) women and 16 (41%) were men with an average age for women 27.1+5.7 years and for men, 26.1+5.2 years.

### Maximum habitual intercuspation (MHI)

It was observed that the overall average of the population studied to MHI in temporal muscles expressed in microvolts (uV) showed 230.1 microvolts for the muscles left and 225.4 microvolts on the right. In the mean values encountered during mastication, 28.0 microvolts were observed for the left side, while the right showed 27.3 microvolts, which resulted in percentage of use of the muscle fibers with average 15.5% to the left and 14.6% right (Table 1).

**TABLE 1:**
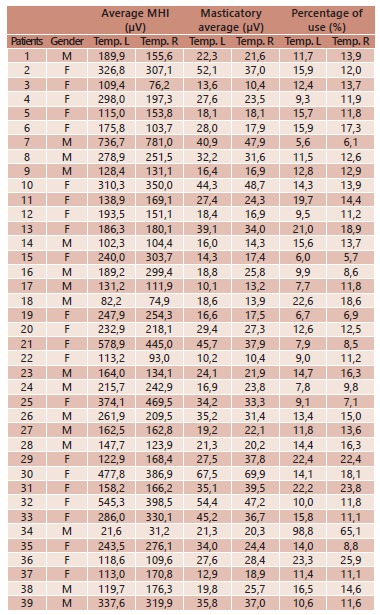
Average values MHI, masticatory function and percentage of use during mastication of the left temporal muscle and right

The overall average for the MHI in masseter muscles expressed in microvolts, showed 157.2 microvolts to the muscles on the left and 181.6 microvolts right. The average values found during mastication were observed 22.8 microvolts to the left side, while the right showed 24.9 microvolts, which resulted in percentage of use of the muscle fibers with average 20.0% to the left and 21 2% right (Table 2).

**TABLE 2:**
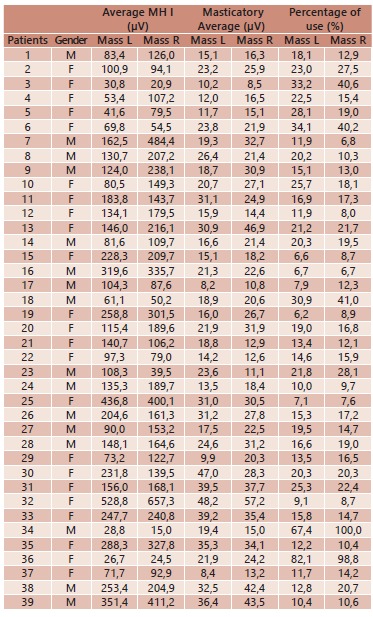
Average values MHI masticatory function and usage percentage during mastication of the left and right masseter muscles

### Percentage of use (PU)

When there is the presentation of the figures for the genders in left and right temporal, proved to be average, minimum and maximum (Table 3), and the categorization of PU muscle fibers required for the exercise during mastication.

**TABLE 3:** MIH values, chewing and usage percentage with differentiation between gender to temporal muscle

Variable			Avg.	Min	Max	DP
MHI (µV)	Temporal L	Women	248,1	109,4	578,9	+137,9
	Temporal L	Men	204,3	21,6	736,7	+162,1
	Temporal R	Women	238,2	76,2	469,5	+116,2
	Temporal R	Men	206,9	31,2	781,0	+172,3
Mastication (µV)	Temporal L	Women	31,4	10,2	67,5	+15,0
	Temporal L	Men	23,1	10,1	40,9	+8,5
	Temporal R	Women	29,5	10,4	69,9	+14,1
	Temporal R	Men	24,2	13,2	47,9	+9,2
Percentage of use (%)	Temporal L	Women	13,8	6,0	23,3	+5,2
	Temporal L	Men	17,9	5,6	98,8	+21,9
	Temporal R	Women	13,5	5,7	25,9	+5,4
	Temporal R	Men	16,3	6,1	65,1	+13,4

In women, the results were for the left side 47.82% (n=11) with PU between 11-20%; 34.79% (n=9) between 0-10% and 17.39% (n=4) between 21-30%. In the right side muscles, 65.21% (n=15) with PU between 11-20%; 21.74% (n=5) between 0-10% and 13.05% (n=3) between 21-30%.

In men left lateral muscles 56.25% (n= 9) presented the PU between 11-20%; 31.25 (n=5) 0-10% and 6.25% (n=1) with PU 21-30% and 6.25% (n=1) between 91-100%. In the right side muscles there was 75.00% (n=12) with PU between 11-20%; 18.75% (n=3) between 0-10% and 6.25% (n=1) between 91-100%.

Introducing the values for the genders in left and right masseter, proved to be average, minimum and maximum (Table 4), and the categorization of PU muscle fibers required for the exercise during the mastication.

**TABLE 4:** MIH values, chewing and usage percentage with differentiation between gender to masseter muscles

Variable			Avg.	Mín	Max	DP
MHI (µV)	Masseter L	Women	162,7	26,7	528,8	+127,4
	Masseter L	Men	149,2	28,8	351,4	+90,8
	Masseter R	Women	178,5	20,9	657,3	+141,0
	Masseter R	Men	186,1	15,0	484,4	+130,7
Mastication (µV)	Masseter L	Women	23,7	8,4	48,2	+11,9
	Masseter L	Men	21,5	8,2	36,4	+7,4
	Masseter R	Women	25,4	8,5	57,2	+11,7
	Masseter R	Men	24,3	10,8	43,5	+9,8
Percentage of use (%)	Masseter L	Women	20,6	6,2	82,1	+15,6
	Masseter L	Men	19,0	6,7	67,4	+14,3
	Masseter R	Women	21,0	7,6	98,8	+19,1
	Masseter R	Men	21,4	6,7	100,0	+22,7

In women the results to the left side were 43.48% (n=10) with PU between 11-20%; 26.09% (n=6) between 21-30%; 17.40% (n=4) 0-10%; 8.70% (n=2) between 31-40% and 4.33% (n=1) between 81-90%. On the right side muscles, 47.83% (n=11) with PU between 11-20%; 26.09% (n=6) 0-10%; 13.05% (n= 3) between 21- 30% and 4.33% (n=1) between 91-100%.

In men left lateral muscles were 56.25% (n=9) with PU between 11-20%; 25.00% (n=4) between 0-10%; 12.50% (n=2) between 21-30% and 6.25% (n=1) with PU 61-70%. In the right side muscles there were 50.00% (n=8) with PU between 11-20%; 31.25% (n=5) between 0-10%; 6.25% (n=1) between 21-30%; 6.25% (n=1) between 41-50% and 6.25% (n=1) between 91-100%.

**FIGURE 1:**
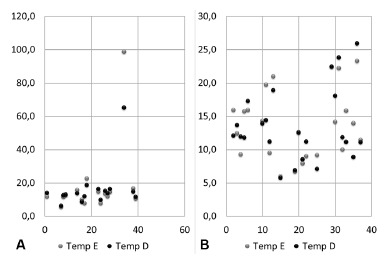
A) Design by the use of temporal muscles of the male group; B) design by the use of temporal muscles of the female group

In Figures 1A and 1B, addressing the results of patients in the respective gender in their left and right temporal muscles, was observed linear plot, with presentation ranging from 6.1 to 22.6% for men and 5.7 to 25.9% of use for women. There was the exception in values in a single person with poor electrical muscle condition in the left temporal, 98.8% of muscle fibers to the masticatory exercise and 65.1% of them to right temporal.

**FIGURE 2:**
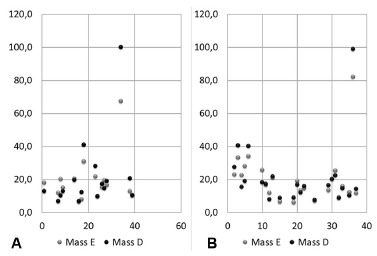
A) Design by the use of masseter muscles of the male group; B) design by the use of masseter muscles of the female group

In Figures 2A and 2B, addressing the results of patients in the respective gender in the left and right masseter muscles, was observed a very well defined linear line, with presentation ranging from 6.7 to 41.0% for men and 7.6 to 40.6% of use for women, but also presenting exception.

In these figures, there were also an exception in their values, coinciding with the same patient of Figure 1A, featuring in the left masseter the value of 67.4% and in the right, reaching 100% the use of fibers for chewing.

For women, one patient had the worst electrical muscle condition left masseter, 82.1% and right masseter, 98.8% for the same performed function.

## DISCUSSION

It is known that the electrical activity is a personal, untransferable number, represented by the value of 100% for the maximum load of each individual collection. By establishing this maximum and turn it into PU got the realization of the fact that patients, when analyzed in their use percentage for chewing, found a range of concentrations involving 11-20%, a fact which allows the comparison between groups without destroying the only collected value of each individual.

On the presented results is evident the largest number of patients in electrical activity of muscle fibers in usage percentage ranging between 11-20%, coinciding this value in both genders.

Research corroborates the idea that the average muscle activity, collected by surface electromyography, increases in proportion to the resistance offered by food for mastication^8^ ^,^ ^10^ ^,^ ^11^ ^,^ ^12^ ^,^ ^17^ ^,^ ^25^. It was also observed that there is variation in the masseter muscle activity values according to the volume of food administered^4^.

Studies have reported the approach to the use of EMG'S in speech therapy in patients with dentofacial alterations that demonstrated a reduction of electric potentials during the masticatory act, reduced the maximum contraction strength and reduced performance of the muscles involved in the mastication function^13^ ^,^ ^24^.

Study^19^ found that the average difference between masseter (right and left) during maximum intercuspation, was 20.0 microvolts and mastication, 10.3 microvolts.

In all these findings we looked for the existence of several studies to the measurement of electrical activity in both population without symptoms and dysfunctions such as in dental diseases; however, it did not reach any data that make correlation between morbidly obese patients and their muscular performance during mastication, as proposed in this study. One can even mention that the methods are different, making it difficult to compare results so that you can draw something around the normal or presentable in a population with morbid obesity.

These data reinforce the characterization of muscle exercise improvement for realization of mastication both the preparation and the benefit of evolution in the postoperative period of gastroplasty. The present results should open space to further studies related to the genesis, maintenance and therapy, searching for effective masticatory function, both in the surgical treatment of obesity as in maintaining the weight lost in the medium and long term.

## CONCLUSION

Between 40-50% of the samples there was electrical activity in masseter and temporal muscles with variable values, and that, after transformation of this unique and intransferable value into a percentage value, facilitated the comparison of the electrical activity load used between the groups, as well as obtained percentage of use of muscle fibers involving 11-20% values. This result is a track that can be called as a reference for the study group. Gender did not constitute variable in morbidly obese patients.
